# Travelling Editorials

**Published:** 1843-05

**Authors:** 


### THE WESTERN JOURNAL.

Vol. VII.—No. V.

LOUISVILLE, MAY 1, 1843.

### TRAVELLING EDITORIALS.

It is no doubt known to most of our readers, that Pensacola Bay is a permanent Navy station, and the only one west of Cape Florida. Having for its object the protection of our commerce on the Gulf of Mexico, it is of special interest to the people of the valley of the Mississippi, and the little sisters—Pearl, Alabama, Escambia, Appalachicola, and Suwana, which enter the Gulf to the east of the great river. We shall not, then, apologize for making our visit to this station the occasion of an editorial; although it may embrace some things not strictly professsional, and others with which many of our readers are already acquainted.

### *The Bay.*

What is now called Pensacola Bay, was discovered, three hundred and four years ago, by one of the followers of De Soto, by whom, however, it was not visited. It was then, by the Indians who dwelt on its banks, called Achusi. Its width varies from one to six or eight miles, and its length is about thirty. Lying in the latest tertiary formation, its banks are composed of white and yellow sand.

The long leaved pine is the predominant tree of the forests which surround it, interrupted, here and there, by a cypress and titi (*andromeda*) swamp. The traveller may wander far and wide, without meeting with a more beautiful sheet of water than that on which we are now looking out, from the verandah of the Naval Hospital, in one of whose untenanted wards we lodged last night. Our latitude is a little above the thirtieth degree—our elevation thirty feet over the Bay.

*Navy Yard.*

This *depot* of munitions of war and naval supplies, is situated on Tartar point, which projects into the north side of the Bay, about three miles from the open Gulf. This point is a bed of white sand, elevated but a few feet above the waters of the Bay. It is said to be an exceedingly healthy spot. Of its buildings, it is not our purpose to speak. Its present commandant is Com. Lavalette; its surgeon Dr. W. A. Spottswood. The officers of the yard are changed every three years.

*Forts.*

Within sight of the yard, are three forts, two at the mouth of the Bay, on the approximate extremities of Foster's Island and Santa Rosa Island, which are but dunes of white sand, rising a few feet above the tide. The former is called Fort McCree, the latter Fort Pickens. The third, Barrancas, is situated on a higher bank, two miles up the Bay, on its north side. A part of the 7th Regiment of Infantry under the command of Lieut. Col. Whistler, and having Dr. Moore as surgeon, is stationed at these forts. Their object is the protection of the Navy Yard, and of vessels undergoing repair.

*Hospital.*

The site of this edifice, is between the Navy Yard and Fort Barrancas. The buildings stand on a second bank or higher terrace, twenty-four feet above the level of the Bay, and are almost concealed by live-oaks, pines, and other evergreens; clumps and tufts of which partially obscure the white sand of the plain below. At the level of this plain, in front of the Hospital, several copious springs of soft water burst out, which form basins and then flow off to the Bay in perpetual currents. In the rear, and on each flank of the Hospital, is a plain of yellow sand, over-shadowed with shrubs and trees of perpetual verdure; and are already, maugre a most inclement and tardy spring, in some degree enamelled with flowers.

The Hospital buildings consist of two principal edifices, connected at their ends by a corridor; of a smaller one in the rear; and of houses for the surgeon and assistant surgeon, in a line with the central structures. Each of these is a parallelogram, surrounded by a broad and high verandah or porch, with an elevated basement. The wards for seamen consist of transverse sections, each sufficient to receive from eight to ten patients; and are admirably ventilated by means of opposite windows. The edifice appropriated to sick officers and midshipmen, has an entry through its centre, with small rooms on each side, which cannot be as well ventilated as the seamen's wards. This entry is too narrow, which results from the inordinate width of the porticos. In fact, the parallelograms might have been made several feet wider, without approaching too near the surrounding colonnades; whereby their capacity would have been much increased at a very small expense. At present there are but nine patients in the establishment. It can accommodate 120, and has been filled to overflowing; which is likely to be the case whenever ships of war arrive infected with yellow fever. Indeed, experience has shown that this is little else than a Yellow Fever Hospital. When the disease of a seaman assumes an exceedingly chronic form, and he is not very ill, or when he has become permanently infirm, he is sent round to the Naval Asylum near Philadelphia. The term of service at the hospital is three years. The present surgeon is the amiable and intelligent Doctor Isaac Hulse, who was present at the founding of the hospital in 1824, and, from requiring, through constitutional infirmity, a warm climate, has been sent here oftener than all the other surgeons of the Navy. At present he has no assistant. Like the other surgeons of the Navy he is allowed to engage in private practice, and is often called to Pensacola, eight miles up the Bay, where he is greatly esteemed for his skill and urbanity.

*Navy Surgeons.*

As many of our young readers may aspire to places in the Navy, we proceed to say something for their benefit. At present there are 69 surgeons, 60 assistants, and 9 passed assistants. The last are those who stand ready for promotion. To obtain admission into the

Navy, the applicant must first be a graduate, and then undergo a successful examination by a board of surgeons. It often happens that the candidate is rejected! If this occurred still oftener, it would be still better. In private life, people may choose for themselves, and so great is the popular propensity to patronize quacks and dunces, that if thoroughly educated graduates were always offered, they would perhaps be rejected. The Government, however, owes it to our brave and patriotic seamen and officers, exposed to all climates and casualties, to provide for them competent physicians and surgeons. It is due, also, to the character of the nation that it should send out such as will bear a favorable comparison with the surgeons of France and England, who, in times of peace, are frequently brought into communication with ours. As long, then, as our medical schools (without a single exception) maintain their present degraded and contemptible standards of medical attainment, their short sessions, and superficial teachings, it is absolutely necessary to have a naval board of examination—to turn back to the horn-books of the profession, one-third of all who apply. Not having been among this number, the candidate receives a certificate, when he may, if a vacancy exist, obtain an appointment from the Secretary of the Navy. But let him be aware that he is only admitted to probation. At the end of five years, he is subjected to a second and still severer ordeal, the great object of which is, to discover what his improvement has been since the former—whether he has been observing and studious, is a growing professional man, and promises to become eminent—if so, he is made a passed assistant, and ready in due time to become a surgeon. Thus, our young friends will see it is no child's play to get into the Navy—with whatever facility they may have crept into the profession, or may creep into professorships.

Let us now inquire into the motives and advantages of a Navy appointment. A naval surgeon is exposed to the dangers of all climates and localities, and necessarily separated, for long periods, from his friends and family; but, on the other hand, he has diversified opportunities of improvement, pleasant companions, and exemption from the caprices and delinquencies which so often annoy or disappoint him in private practice, and derives importance and dignity from his official rank. But all this—

"Plays round the head, but comes not to the heart."

The great American question is, which career will fetch in most money? As data for an answer to this momentous inquiry, we shall extract from the Naval Register of our friend Hulse, the following statement of the pay of surgeons and assistants, in 1842.

"Assistant surgeons, waiting orders, $650 *per annum*—at sea $950. Passed Assistants, waiting, $850—at sea $1200—when stationed at a navy yard, hospital, rendezvous, or receiving ship, $950. Surgeons, for the first five years after being commissioned, $1000; for the second five years $1200; for the third $1400; for the fourth $1600; after the 25th year $1800. All surgeons under orders for duty at navy yards, hospitals, or receiving ships, to have an increase of one-fourth of the above amounts; if ordered to sea, of one-third; and if sent out as fleet surgeons, one-half. The Chief of the *Bureau of Medicine and Surgery*, at Washington, has a salary of $2500." From an examination of this table, we incline to the opinion, that if they were all in actual service, the 138 medical gentlemen of the navy, would receive as much as a corresponding number of physicians, taken indiscriminately in any part of the United States out of our largest cities; but it must be admitted that there are many in the navy, who could earn more in private practice.

### *Navy Ration.*

Having got out of port and fairly under weigh, we shall not cast anchor till we have sailed a little further. We suppose that most of our backwoods' readers have not had an opportunity of knowing on what our seamen subsist, and we will, therefore, proceed to tell them.

The *daily* allowance of each person is—"One pound of salted pork, with half a pound of peas or beans; or one pound of salted beef, with half a pound of flour, and a quarter of a pound of raisins, dried apples, or other dried fruits; or one pound of salted beef with half a pound of rice, two ounces of butter, and two ounces of cheese, together with fourteen ounces of biscuit, one-quarter of an ounce of tea, or one ounce of coffee, or one ounce of cocoa; two ounces of sugar and one gill of spirits; and a *weekly* allowance of half a pound of pickles or cranberries, half a pint of molasses, and half a pint of vinegar."

"Fresh meat may be substituted for salt beef or pork, and vegetables or saur kraut, for other articles usually issued with the salted meats; allowing one and a quarter pounds of fresh meat for one pound of salted beef or pork, and regulating the quantity of vegetables or saur kraut, so as to equal the value of those articles for which they may be substituted."

"Should it be necessary to vary the above daily allowance, it shall be lawful to substitute one pound of soft bread, or one pound of flour, or half a pound of rice, for fourteen ounces of biscuit; half a pint of wine for a gill of spirits; half a pound of rice for half a pint of peas or beans; half a pint of peas or beans for half a pound of rice,"

No officer or midshipman, nor seaman under 21 years of age, is allowed to draw the spirit part of the ration; and any one may commute it for money.

It must be admitted, that we have travelled over a substantial bill of fare; and that our seamen are better fed than many of their brothers on shore. The diversity in their diet is highly commendable, and may be regarded as a main reason why they are so seldom affected with scurvy.

### *Waste of Hospital Liquors.*

In the office of the Hospital where we are writing, we find conspicuously pasted against the wall, a circular from Dr. W. P. C. Barton, Chief of the Bureau of Medicine and Surgery, in which he sets forth, that great abuses have existed in the care and use of the liquors provided for the sick; that they have been considered by the officers as a store from which all might borrow, and that payment was generally omitted; that they have been wasted on the well, or on those who did not need them; that it will hereafter be considered a misdemeanor to lend even a glass of wine; and that he believes the necessity of liquors in the treatment of the sick is greatly overrated. It was not without a feeling of mortification that we read an official statement, from such high authority, which presents our naval officers under such a discreditable aspect, and we fervently hope the Chief of the Bureau may be successful in reforming the abuse he has made known to the world.

### *Assault on the Temperance Reform.*

The circular from which we have just quoted, concludes as follows:

"Although the undersigned has not been able to perceive anything but future fatal reaction from the high handed ultra temperance cause so injudiciously, as he believes, expanded beyond its first design, and of course belongs to no temperance society, still the rational community is not entirely swallowed up in the mad vortex. Hence the moral aspect of the subject, in unison as it is with the popular feeling of the judicious temperance advocates of the present world, does not take any thing from the merits of a necessary reform."

Now we cannot but deeply regret that the Chief of the Bureau at Washington, should have so far stepped out of the path of official duty, as to put forth this denunciation.

What is it, we would ask, that led to the causes he has so vividly depicted, but an overweening love of drink, prompting to an unscrupulous seizure even of all provided for the sick? And what so likely to correct the evil, as to render drinking unfashionable and odious? Why has the government abolished the army ration of spirits, and reduced that of the navy one-half, if drinking is *necessary?* If not necessary for soldiers, why is it necessary for people in private life? If soldiers are not allowed alcoholic drinks, nor suttlers allowed to sell them, the army, in theory, is a *total abstinence* temperance society; but according to Dr. Barton, this is fanatical, and if so, the spirit ration ought to be restored. Does he not know that total abstinence is the best method of moderating the desire for drink; and that the "gill a day" for the navy is a means of keeping alive the propensity? He tells us, that the "*rational* community is not *entirely* swallowed up in the mad vortex." This is true. There are a *few* rational friends of temperance who have not been drawn into it, but the number is very small. The "mad vortex" has spread itself over the whole country; and is not likely to contract, notwithstanding the Doctor sees nothing ahead but "future fatal reaction." He deprecates the "high handed" proceedings of the friends of temperance! But what are they? Is it a "high handed" proceeding, for a man to resolve and declare that he will not again lift the intoxicating bowl as high as his mouth? Is it "high handed" for a man to decline drink, when it is offered to him? Is it "high handed" to neglect to ask others to drink? Is it "high handed" to persuade our officers and seamen not to rob the sick of the wine provided for their relief? Is it "high handed" for a physician to tell the people, of whom he is the medical adviser, that they would have better health if they did not drink at all? Is it "high handed" for us to ask the Doctor, whether every seaman and officer of the navy would not have firmer health, greater strength and activity, a milder temper, and a more willing heart, if he should never again taste a beverage more stimulating than tea and coffee? Is it "high handed" for benevolent men to address the intemperate, and implore them not to lay waste their habitations and beggar their wives and children? If it be, then the friends of the temperance reform *are*, indeed, "high handed;" and we trust will continue so forever. D.

*Navy Hospital, Pensacola Bay,*
March 30th, 1843.

